# The generation of marine litter in Mediterranean island beaches as an effect of tourism and its mitigation

**DOI:** 10.1038/s41598-020-77225-5

**Published:** 2020-11-23

**Authors:** Michaël Grelaud, Patrizia Ziveri

**Affiliations:** 1 Institute of Environmental Science and Technology (ICTA), Universitat Autònoma de Barcelona (UAB), Bellaterra, Spain; 2 Catalan Institution for Research and Advanced Studies (ICREA), Barcelona, Spain

**Keywords:** Environmental impact, Sustainability

## Abstract

The Mediterranean Sea and its coastal systems are threatened by intense anthropogenic pressures including rapid accumulation of marine litter by diverse human activities. The region, which is the world’s leading touristic destination, has to face a seasonal increase of waste generation due to the seasonal influx of visitors. The beaches, extremely crowded during the summer, are particularly vulnerable since they are proven to be concentrated accumulation zones and one of the main gateways of litter to enter the marine system. We found that the accumulation rates of marine litter on Mediterranean island beaches follow a seasonal pattern, increasing up to 4.7 times during the high season, representing a daily load of (40.6 ± 11.5) 10^6^ items/day extrapolated to all the islands of the region. We developed an accumulation index to assess the dynamics of marine litter and support efficient mitigation strategies by local authorities. To limit marine litter production attributable to recreational activities, a series of pilot actions implemented during the high touristic season, demonstrated a substantial reduction (up to 52.5%). The implementation towards an efficient and sustainable tourism business model is urgently required.

## Introduction

“Any persistent, manufactured or processed solid material discarded, disposed of or abandoned in the marine and coastal environment” is considered as marine litter^1^. Revealed during the late 1960s, the marine litter issue has received growing attention from the public^2^ with for example the foundation of the International Coastal Cleanup Programme in 1986^3^ and culminating in the 2000s with the recognition of the microplastics (MPs) issue^4^ or the recent attempts to clean the oceans (e.g. The Ocean Cleanup Project^5^). Despite the different approaches implemented to tackle this contamination and which can be divided into four main categories^6^ (preventive, mitigating, removing and behavior-changing), the yearly amount of litter reaching the marine environment and mainly driven by plastic pollution remains tremendously high^7,8^.

With a coastal population of nearly 150 million inhabitants, the influx of freshwater from densely populated river catchments and a contribution to 15–30% of the global shipping activity^9,10^, the Mediterranean Sea has been recognized as one of the most affected areas in the world by marine litter^11^. In addition to these stressors, the countries surrounding the region yearly attract about one third of the world tourism^12^. Taken together, these anthropogenic pressures make this semi-enclosed sea, characterized by an anti-estuarine circulation^13^, an accumulation zone for marine litter, with for example concentrations of floating plastics debris comparable to those observed within the five subtropical gyres^14–16^. This high contamination of the Mediterranean Sea goes hand to hand with a stream of adverse effects to marine ecosystems, public health or socio-economic costs^11^.

At a global scale, beaches are one of the main land-based sources for litter to enter the marine environment through inadequate waste management, littering and illegal dumping^17,18^. The Mediterranean Sea is not an exception^11^, particularly during the summer: the beaches of *Mare Nostrum* are a hotspot for leisure, especially for those who are looking for the three S’s—sea, sand and sun^19^. Although beneficial for the sustainability of local communities, tourism may have detrimental socio-economic and environmental impacts^20^. This is particularly true for sea locked areas such as the islands of the region, which due to their attractiveness will host a far greater population during the summer^21^. In particular, coastal municipalities have to adapt and cope with the increase of the waste generated, including on the beaches, by the seasonal inflow of tourists^22^.

The cleanliness of the beaches being one of the main factors considered by the tourists, along with the scenery, the safety, the facilities and the water quality^23^, marine litter can represent, in addition to its negative environmental effects, a shortfall^24^ for local economies which often depend on this financial windfall. For example, in many countries, including those from the Mediterranean region, the absence of litter dictates visitors’ choice (see Ref.^25^ and references herein) and the probability to return to a given beach is strongly associated to the quality of the coastal environment^26^.

In this general context, it is then crucial to understand the seasonal dynamics of the waste generated by the visitors on the beaches in order to design effective solutions to mitigate this issue and move toward more sustainable tourism. Here we present the results of marine litter monitoring surveys performed on 24 beaches, going from remote to highly touristic sites, of Mediterranean islands during both the low and high touristic season of 2017. The results are compared to monitoring surveys performed on 11 of the same sites in 2019 after the implementation of pilot actions aiming at reducing the waste generated by the visitors.

### Seasonal variation of beach litter in 2017

**Figure 1:**
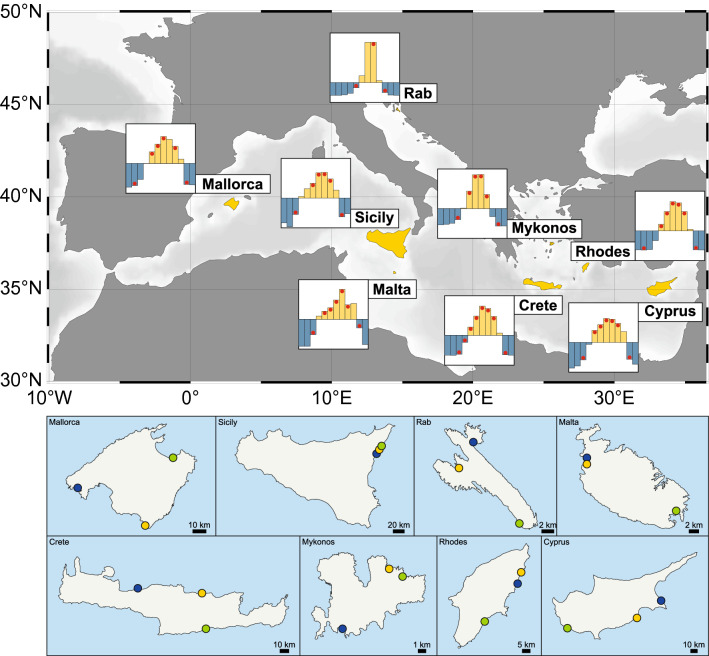
General map of the Mediterranean Sea. Top: the 8 islands selected for this study. The graphic next to each island shows the normalized monthly number of tourists (Y axis, arbitrary scale) welcome in 2016 or 2017 (X-axis, from January to December) (Supplementary Table S1). Yellow bars: high touristic season (monthly number of tourists above the annual average), blue bars: low touristic season (monthly number of tourists below the annual average). Red dots: marine litter surveys. Below: map of each island with the selected beaches (T_beach_: yellow dots, L_beach_: blue dots and R_beach_: green dots). The general map of the Mediterranean Sea was generated with Ocean Data View (Schlitzer, R., Ocean Data View, https://www.awi-bremerhaven.de/GEO/ODV, 2001).

In order to evaluate the seasonal variation of marine litter as an effect of tourism on sandy beaches of Mediterranean islands, 147 surveys were conducted in 2017 during both the low and high touristic season, from February to November. For each of the eight participating islands (Mallorca—Spain, Sicily—Italy, Rab—Croatia, Malta—Malta, Crete, Mykonos and Rhodes—Greece, and Cyprus), three different beaches were selected: a very touristic beach (T_beach_), a beach mainly used by locals (L_beach_) and a remote beach (R_beach_) with limited use by humans (Fig. 1 and “Methods”). For each beach, a periodic monitoring was performed on the same fixed 100 m portion^27^, covering the area going from the water line to the back of the beach^28^ (Supplementary Fig. S1). Here, any item found was collected, characterized and properly disposed of. We included the smaller size classes of mesoplastics (MePs, 0.5–2.5 cm), large microplastics (MPs, 0.1–0.5 cm; i.e. visible to the naked eye), as well as pellets (raw plastic material), which usually are not targeted during this type of survey^27^.

**Figure 2:**
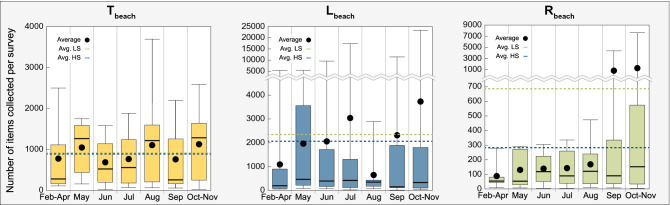
Number of items collected. Box plots showing the number of items collected on average per 100 m of beach (T_beach_ in yellow, L_beach_ in blue and R_beach_ in green). Note that the R_beach_ from Malta was excluded (see “Methods”). Boxes represent the limits of the first and the third quartile. The line inside the boxes is the median and the extended lines are the maximum and the minimum number of items collected (note the broken Y axis with a different scale for L_beach_ and R_beach_). Black dots: average number of items collected, green dashed line: average number of items collected during the low season (LS) and blue dashed line: average number of items collected during the high season (HS).

A total of 162,320 items were collected. After correction for the distance and excluding one beach (see “Methods”), this represents an average of 1197.6 ± 2978.0 items per survey (or per 100 m of beach), 526.9 ± 794.2 items if we exclude the plastic fragments of less than 2.5 cm. These results fall in the same order of magnitude as others studies carried out on Mediterranean Sea beaches^11,29–34^. The vast majority of the items collected belong to the artificial polymer materials (94.2%; Supplementary Fig. S2), i.e. items partly or completely made of plastic. The average number of items collected per survey depends on the type of beach considered: the L_beach_ has an average of 2157.2 ± 4680.2 items/100 m (n = 49), followed by the T_beach_ 899.9 ± 858.8 items/100 m (n = 49) and the R_beach_ 425.4 ± 1319.9 items/100 m (n = 42) (Fig. 2). Interestingly, when only the number of items collected is considered, no clear seasonal variation can be observed, no matter the type of beach considered (Fig. 2). Moreover, the number of items collected per 100 m of beach is always higher during the low season compared to the high season (× 1.03 for the T_beach_, × 1.14 for the L_beach_ and × 2.31 for the R_beach_). These first results suggest that the seasonal increase of visitors on the beaches does not affect the amount of items of litter. This is counter intuitive as, for example, the amount of municipal solid waste generated during the high season on the island of Menorca (west Mediterranean Sea, Spain) is directly related to the number of tourists^35^.

For a given beach, by taking into account the time elapsed between the monitoring and the last cleaning performed by local authorities for the collected items larger than 5 mm, the time elapsed between two consecutive monitoring for the items smaller than 5 mm (i.e. MPs and pellets), the surface monitored and the number of items collected, it is possible to estimate the accumulation rates (AR) of the marine litter (see “Methods”). During the high touristic season, the monitoring was done on average 6.2 days after the last cleaning for the T_beach_, 13.2 days for the L_beach_ and 27.8 days for the R_beach_, while during the low season, it was done respectively on average 61.2 days, 79.0 days and 105.3 days after. For some beaches, no cleaning activity was performed by the local authorities. In this case, we used the time elapsed between each monitoring to calculate the AR.

**Figure 3:**
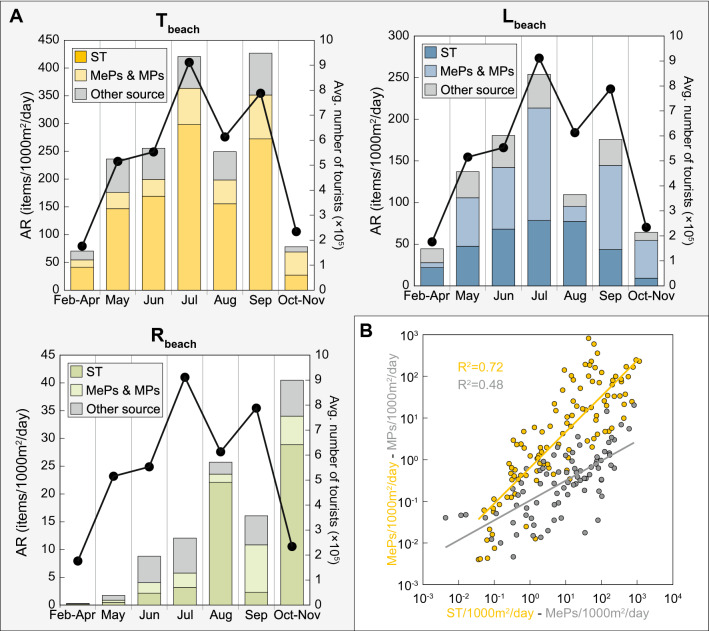
Accumulation rates of marine litter by source. (**A**) AR over time of the items from the “shoreline, including poor waste management, tourism and recreational activities” category (ST, dark color), the MePs and MPs (light color) and all the others categories (grey color). The black dots and lines represent the average monthly number of tourists welcomed in the islands where monitoring was performed (Supplementary Table S1). The decrease observed during August for the AR of the T_beach_ and the L_beach_ as well as for the average number of tourists is related to the absence of monitoring performed on the island of Mallorca, which is the most visited island and one of the most affected by marine litter. (**B**) Comparison between the AR of the MePs and the AR of the items from ST category (yellow); and between the AR of the MPs and the AR of the MePs (grey). The solid yellow and grey lines represent the power regression for both comparisons (both X and Y axes are on a logarithmic scale).

When the ARs are considered, a completely different picture emerges: the AR calculated for the three types of beach show an increase during the high touristic season (Fig. 3a). The T_beach_ has the highest accumulation with an average of 329.6 ± 444.9 items/1000 m^2^/day (n = 33) during the high season, followed by the L_beach_, 177.2 ± 265.3 items/1000 m^2^/day (n = 33) and the R_beach_, 13.7 ± 32.2 items/1000 m^2^/day (n = 28). This represents an increase of the AR of + 471.9% for the T_beach_ and + 346.0% for the L_beach_ compared to the low season, when the AR are respectively of 69.8 ± 110.1 items/1000 m^2^/day (n = 16) and 51.2 ± 101.2 items/1000 m^2^/day (n = 16). For the R_beach_, the high season increase is concealed by the high AR recorded in October–November (Fig. 3a). The ARs for this specific period fall in the same range as for the T_beach_ and the L_beach_. This could suggest that the impact of the visitors on the R_beach_ during the high season is lower than the “natural” beaching of the litter during the low season, which is usually characterized by worse weather conditions. This could possibly result in an increased wash up of the marine litter onto the beaches, even if very little is known about the processes that govern its beaching^36^. Then for the R_beach_, although a seasonal pattern is perceptible, the AR during the high season [13.7 ± 32.2 items/1000 m^2^/day (n = 28)] are on average 32.7% lower compared to the low season [20.3 ± 59.7 items/1000 m^2^/day (n = 14)].

To our knowledge, very few studies provide the necessary information to estimate the AR of marine litter on beaches^37–41^. Three of them were carried out on islands with very low anthropogenic pressure compared to our study: on a remote Aleutian island^37^, a remote island of the Hawaiian archipelago^40^ and a remote sub-Antarctic island^38^. In each case, the estimated ARs are up to 2–4 orders of magnitude lower than on the Mediterranean islands beaches, and up to 1–2 orders of magnitude lower if we consider beaches from the Mediterranean Sea^34^. For the study performed on a beach of eastern Australia^39^, the estimated ARs fall in the same range, highlighting the relationship between the number of items collected and the time elapsed since the last cleaning/monitoring.

### Most frequent items and MePs–MPs possible source

Once corrected for distance, in order to make all the selected sites comparable (see “Methods”), the five most frequent items collected, were the MePs (35.3%), cigarette butts (12.4%), the MPs (11.2%), pellets (9.5%) and macroplastics (> 2.5 cm; 8.3%). The possible sources were classified according to the eight major categories described for the Adriatic and Ionian seas^27^. When we look at the items from the “shoreline, including poor waste management, tourism and recreational activities” (ST category, i.e. the items most likely left on the beaches by the visitors), the five most frequent for the surveys of 2017 are cigarette butts (12.4%), plastic caps and lids (3.5%), cutlery, trays and straws (1.6%), crisp, sweet packets and lolly sticks (1.4%) and metal bottle caps (0.7%); in agreement with previous studies^29,30^. During the high season, these five items present as well the highest AR of the ST category no matter the type of beach considered, with the exception that for the R_beach_, the cutlery, trays and straws are replaced with metal drink cans (Supplementary Fig. S3). Unsurprisingly, cigarette butts have the highest AR with values of 173.5, 41.3 and 1.6 items/1000 m^2^/day measured respectively at the T_beach_, the L_beach_ and the R_beach_ during the high season.

Since the origin of small fragments/pieces of plastics cannot be clearly defined, we attributed the pellets to the “shipping” category, meaning that they are lost at sea during transportation. The MePs and the MPs are usually assigned to the “non-sourced” category. However, we observed a strong correlation between the AR of the MePs and the AR of the items from the ST category (R^2^ = 0.72, n = 116, *p* < 0.001), as well as between the AR of the MPs and the AR of the MePS (R^2^ = 0.48, n = 87, *p* < 0.001) (Fig. 3b), suggesting that the accumulation of small plastic fragments increases with the accumulation of the items of the ST category on the monitored beaches. From this observation, we hypothesize that a non-negligible part of both MePs and MPs could be produced directly on the beaches by the fragmentation of larger plastic items^42^. During the summer in particular, plastic debris on beaches will undergo thermo-oxidative degradation^43^ by solar irradiance, and mechanical degradation^43,44^ by friction with the sand, accelerated by the high volume of visitors (our hypothesis).

Taken together, the AR of the items from the ST category, the MePs and the MPs show a clear seasonal pattern (Fig. 3a). This seasonal variation mimics the average number of tourists welcomed in the participating islands for the T_beach_ and the L_beach_ (Fig. 3a). During each month of the high season, the items from the ST category represent on average 65.7% ± 2.8% (T_beach_), 39.8% ± 17.9% (L_beach_) and 35.5% ± 28.7% (R_beach_) of the items accumulating on the beaches, falling in the same range reported by previous studies^29,34^; and they increase respectively up to 79.2% ± 4.5%, 77.9% ± 9.4% and 58.8% ± 19.1% if the MePs and the MPs are considered. The ARs of the other category items remain quite stable over the year (Fig. 3a). The highest AR for the MePs and MPs observed in the L_beach_ could be explained by the longer time elapsed between the monitoring and the last cleaning, allowing more time for larger plastic debris to fragment.

### Accumulation index

**Figure 4:**
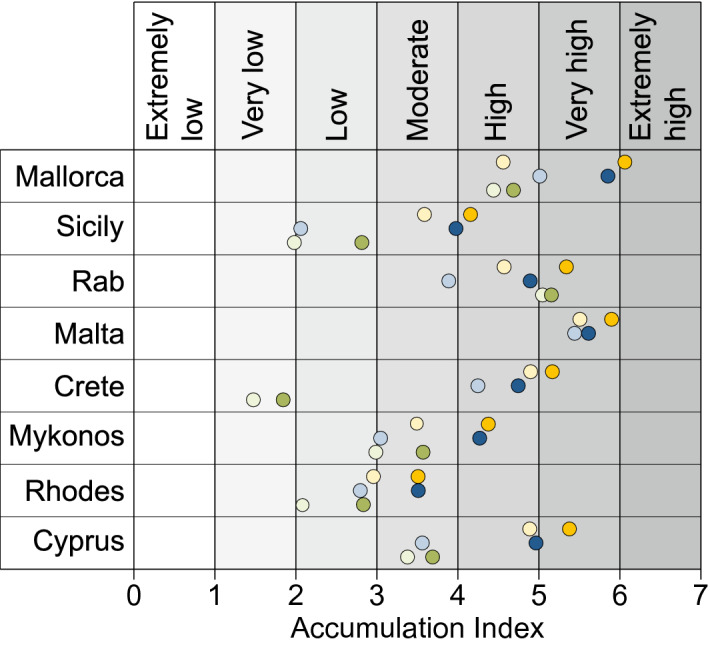
Accumulation Index of the 24 selected beaches. The AI calculated for the low season (LS) are given by the light colors and the AI for the high season (HS) by the dark colors. T_beach_: yellow, L_beach_: blue and R_beach_: green.

We found that the use of a marine litter accumulation index (AI) is critical for assessing the impact of tourism on coastal sites and specifically on beaches. This AI, based on the AR, is divided into seven categories from extremely low to extremely high (Table 1). Contrary to the existing clean coast index^45^, which provides a snap shot of the cleanliness of a beach, the AI enables the assessment of marine litter dynamics. The AI can be applied at different time scales (month or season) and for general or specific items of marine litter. Here we present the marine litter AI, for each Mediterranean island and for each type of beach monitored in this study (Fig. 4). In all cases, the AI is always higher during the high season compared to the low season, no matter the type of beach considered. The AI can be used as an indicator for municipalities to identify, address and mitigate the seasonal variation of waste generation on beaches resulting from tourism, by highlighting for example the most frequent items abandoned and to raise awareness in order to change visitors’ behavior.

**Table 1:** Accumulation Index (AI), value and equivalence for the accumulation rates (AR).

Quality	Extremely low	Very low	Low	Moderate	High	Very high	Extremely high
AI (log10(AR × 1000))	≤ 1	1–2	2–3	3–4	4–5	5–6	≥ 6
AR (#items/1000m^2^/day)	0.01 ≤	0.01–0.1	0.1–1	1–10	10–100	100–1000	≥ 1000

### Positive impact of awareness campaigns on marine litter AR

Following the results obtained in 2017, pilot actions were conducted during the 2019 high season on 11 beaches previously monitored: both the T_beach_ and L_beach_ of Sicily, Malta, Crete, Rhodes and Cyprus; and the T_beach_ of Mallorca. Due to the very low AR measured during the 2017 high season in the R_beach_, no pilot actions were implemented there. The pilot actions consisted mainly in awareness campaigns^46^ through the distribution of flyers and the installation of posters at the main entrances of the beaches showing the results obtained in 2017 and emphasizing the potential role of the visitors on beach litter generation (11 beaches), the availability of ashtrays (6 beaches) and the intervention of representatives informing the visitors of the marine litter issue and its prevention (9 beaches). Moreover, when nonexistent, new trash bins were installed on the beaches and the existent ones, when necessary, were adapted to collect both mixed and recycled waste, with bilingual indications (national language and English) (5 beaches).

The surveys were conducted in August or September 2019, between two weeks and two months after the beginning of the implementation of the pilot activities and on average 6.7 days (T_beach_) and 35.1 days (L_beach_) after the last cleaning of the beaches. The surveys were carried out on the exact same portions of beach as for 2017 and following the exact same procedure. However, it has been only possible to conduct one survey per site. This constraint made impossible to estimate the AR for the MPs and pellets, the smallest items, after the implementation of the pilot actions. With the exception of the L_beach_ of Sicily which shows a more than sixfold increase of the AR of the items from the ST category, the 10 other sites show a decrease of their AR ranging from − 20.8% (T_beach_ of Cyprus) to − 98.8% (L_beach_ of Rhodes) (Supplementary Fig. S4).

**Figure 5:**
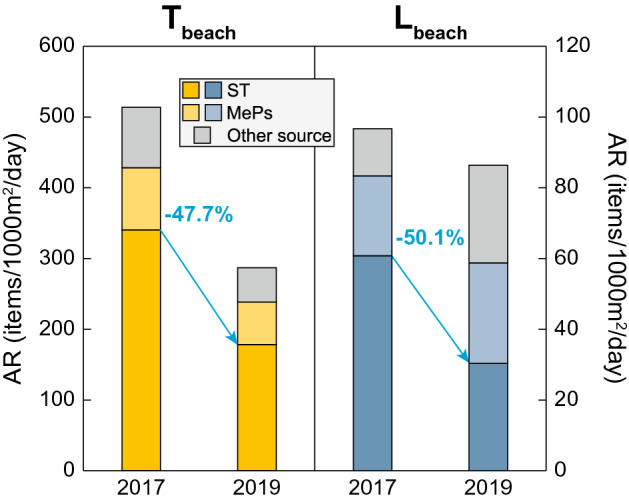
Impact of the pilot actions. AR of the items from the ST category, the MePs and the remaining items (excluding MPs and pellets) measured in August/September2017 and in August/September 2019 after the implementation of the pilot actions. The light blue percentages provide the decrease of the AR for the ST category between 2017 and 2019.

For the 6 T_beach_ tested, the average AR of the items belonging to the ST category were of 340.3 ± 410.7 items/1000 m^2^/day in 2017 and of 178.1 ± 200.2 items/1000 m^2^/day in 2019, representing an average decrease of − 47.7% of the AR. For the L_beach_ tested, after excluding the beach of Sicily (Supplementary Fig. S4), the average AR of the items from the ST category were of 60.8 ± 94.1 items/1000 m^2^/day in 2017 and of 30.3 ± 40.7 items/1000 m^2^/day in 2019. This represents an average decrease of 50.1% of the accumulation of the litter left on the beaches by the visitors (Fig. 5). For the T_beach_ tested, the decrease of the AR of the ST category items is concomitant to a decrease of the AR of both MePs (− 31.3%) and remaining litter (− 43.6%, MPs and pellets being excluded), while for the L_beach_ tested it is concomitant to an increase of the AR of both the MePs (+ 26.0%) and remaining litter (+ 106.7%, MPs and pellets being excluded) (Fig. 5). Regarding the the MePs, the increase of the AR on the L_beach_ could again be explained by a much longer time elapsed between the last cleaning activities and the surveys. However, the difference regarding the other remaining items (i.e. not from the ST category or the MePs), cannot be explained by our results. Although it is possible to imagine that local/regional differences about the beaching of the litter, especially for islands from distinct sub-basins of the Mediterranean Sea, could play a significant role.

According to the results from 2017, cigarette butts are the most common item left on the beaches by the visitors. Ashtrays were then made available for the visitors on the 2 selected beaches of Malta, Crete and Cyprus. The accumulation rates of cigarette butts for these 6 beaches decreased on average by 54.5% (± 12.0%) after the implementation of the pilot actions. For the beaches where no ashtrays were made available (T_beach_ of Mallorca, Sicily and Rhodes; L_beach_ of Rhodes), we observed a decrease of 57.8% (± 28.2%) between 2017 and 2019. No significant difference between the two groups of beaches is observed (*p*-value > 0.05). We do not interpret this as an ineffectiveness of the ashtrays to reduce the number of cigarette butts thrown on the beach, but rather as an increase of the proportion of visitors who possibly refrain from smoking on the beach or who properly disposed of the cigarette butts whether ashtrays are available or not. Interestingly, the only beach which shows an increase of the AR of the cigarette butts in particular (8×) or of the items of the ST category in general (6×) was the L_beach_ of Sicily where no representative was present to inform the visitors and no ashtrays were made available.

Regarding the four other most abundant items, they all show a decrease of their AR on the tested T_beach_: − 51.8% for the plastic caps and lids, − 74.0% for the cutlery, trays and straws, − 59.5% for the crisp, sweet packets and lolly sticks, and − 40.6% for the metal bottle caps. For the L_beach_ considered (excluding Sicily), with an exception for the crisp, sweet packets and lolly sticks which shows an increase of + 153.8% (2 items collected in 2017 vs. 59 in 2019 for these 3 beaches), the other items present a similar decrease of their AR by − 50.7% (plastic caps and lids), − 65.0% (cutlery, trays and straws) and − 42.4% (metal bottle caps) (Supplementary Fig. S5).

### The recreational use of beaches as a main source for marine litter

Massive tourism in Mediterranean islands and specifically in their beaches is a main driver of marine litter generation. Popular beaches (T_beach_ and L_beach_) are clearly the most impacted sites of this study. Every day, during the high touristic season peak (July–August), on every 100 m of beach, visitors will leave on average 844.5 (T_beach_) and 295.2 (L_beach_) items from the ST category. This represent respectively 71.0% and 31.1% of the total amount of items composing the marine litter during this specific period. If the MePs and the MPs are included, resulting from the fragmentation of larger plastic items as we hypothesized, then visitors could generate in total 1028.4 items (T_beach_) and 798.3 items (L_beach_), representing this time respectively up to 86.4% and 84.0% of the total amount of items composing the marine litter during this specific period. Once again it is worth noting that on average 2.7 times more MePs and MPs are generated on the L_beach_ compared to the T_beach_ during the high season peak. To our point of view it is clear that the frequency of the cleaning of the beaches by the local authorities play an important role regarding the prevention of the generation of MPs and MePs as the T_beach_ are cleaned on average 2.1 times as often as the L_beach_. For the R_beach_, tourism could be responsible of the accumulation of 57.7 items per 100 m of beach per day, meaning that the remote sites are on average 14–20 times less impacted than the most popular beaches.

Even if the T_beach_ and L_beach_ are frequently cleaned during the high season, the portion of the marine litter accumulating on these beaches and reaching the sea, although hardly assessable, could represent a tremendous daily load as Mediterranean islands account for about 19 000 km of coastline^47^. At the region scale, we estimated that during the high season peak (July–August), visitors could be responsible for the accumulation of about 40.6 10^6^ ± 11.5 10^6^ items/day on the beaches of the Mediterranean islands (“Methods”). As a matter of fact, if all these items were to reach the marine environment, this would represent the equivalent of a daily accumulation of 2500 ± 709 items/km along the Mediterranean islands coastline. Although hypothetical, this value is quite realistic when compared to concentrations of floating plastic in the nearshore water strip (i.e. < 1 km off the coasts)^48^ ranging from 28,000 to 578,000 items/km^2^.

### Awareness campaigns, a promising tool to reduce beach littering

Our results show that awareness campaigns could be an efficient tool to reduce the amount of litter resulting from the recreational use of the Mediterranean island beaches. Indeed, we observed an average decrease of 52.5% ± 20.8% of the AR of the items from the ST category after the implementation of the pilot actions. These very encouraging results probably benefit from the growing attention of the public to the plastic pollution in the oceans or the measures adopted by the European Commission to reduce marine litter (e.g. the single-use plastic directive). However, this reduction of the marine litter has a price: to achieve these results, the average cost of the pilot actions, extrapolated to the whole high season (May–September) would be of 111.6 k€ per km of beach^49^. This cost could be restrictive for local authorities to implement such actions.

The number of visitors in the Mediterranean region is projected to increase rapidly with more than 500 million expected tourists by 2030^50^. There is an urgent need for a tourism lifestyle change and environmental education of key actors acting on the knowledge gaps of policy-making processes and on the investment in sustainable tourism. Proper monitoring of tourism sustainability is a strategic driver for the economic development and sustainability of islands that depends on ecosystem services already threatened by climate change.

## Methods

### Sampling strategy

In order to assess the seasonal variation of marine litter as an effect of tourism on Mediterranean islands, 24 beaches (3 per island) have been monitored for marine litter during both the low and high seasons. For each selected site, a fixed 100 m portion of beach (i.e. sampling unit) was defined, as recommended in most beach litter monitoring methodologies^27,28,51,52^, and going from the waterline to the back of the beach as the visitors can occupy any point within this zone. Moreover, the sampling unit should be connected, when possible, to at least one access to the beach around which the density of visitors is usually the highest, especially for the T_beach_ and L_beach_. As on some islands the beaches can be very small (< 200 m) and narrow (< 10 m), the monitoring of more than one sampling unit^27,51^ could not be implemented and the monitoring of few (very short) random transects^52^ within one sampling unit possibly not representative. By taking into account all these points, we decided to use the OSPAR methodology^28^ for the 24 selected beaches and adapt it in order to include the MePS, the MPs and the pellets.

Once defined, the fixed 100 m portions have been periodically monitored for marine litter: once a month during the high touristic season (from May to September) and one time before (February–April) and after (October–November) the high season. Although, the same fixed portion was always monitored for each beach, it appears that for some of them, the surface monitored slightly changed from one survey to another, depending on various factors such as the accuracy of the GPS, the presence of vehicles or the crowd on the beach. The highest variations were observed in August and September for the T_beach_ of Malta where only one third of the defined surface was monitored. During the monitoring, all the items with an anthropogenic origin found on the sand were collected, counted and characterized^28^.

During the surveys, the small plastic pieces including the mesoplastics (MePs, 0.5–2.5 cm), the large microplastics (MPs, 0.1–0.5 cm) and the pellets (raw plastic material) were collected at the same time as the larger marine litter items and on the same portion of beach. The surveyors had the instruction to only collect the small items laying directly on the surface of the sand (i.e. plastic debris/pellet buried below the surface of the sand were not monitored). Moreover, during a monitoring, there was one surveyor assigned to the collection of these small plastic items who was helped by the others surveyors once the bigger marine litter items were collected. The small pieces of plastic were then shipped to our institute, sorted according to their size and assigned to one of the following categories: macroplastics (> 2.5 cm), MePS (0.5–2.5 cm), MPs (< 0.5 cm) or pellets. Note that for the pellets, all the particles recognized as such were counted as pellets, no matter their size.

For each island, the 3 different beaches were selected as follows in order to include (1) an impact site (T_beach_) where tourists represent most of the visitors to the beach, (2) a control site (L_beach_) where locals represent most of the visitors and (3) a remote/preserved site (R_beach_) where the frequency of both tourists and locals is low. T_beach_ are characterized by a high volume of visitors, mainly tourists, especially during the high touristic season. In the area situated directly behind the beach high levels of infrastructure dedicated to tourism and recreational activities, such as hotels, restaurants, bars, souvenir shops, etc., are found. L_beach_ are characterized by high volume of visitors, mainly locals, especially during the high touristic season. However, in the area situated directly behind the beach low level of infrastructures dedicated to tourism and recreational activities, such as hotels, restaurants, bars, souvenir shops, etc., are found. R_beach_ are assumed to be the less impacted type of beach. They are characterized by low volume of visitors, even during the high touristic season. In the area situated directly behind the beach no infrastructure dedicated to tourism and recreational activities are found.

Regarding this last type of beach, although 8 beaches were monitored in 2017, we had to remove the results obtained from Malta. The volume of visitors, although reduced, cannot be considered comparable to other remote beaches of this study. It is situated in the municipality of Marsaxlokk and a harbor is bordering the beach to the west. Moreover the total number of items collected on this beach (4249) represents more than 20% of the total number of items collected on all the R_beach_ (20,738), and once calculated the accumulation rates of marine litter for this specific beach appears to be 60 times higher (956.6 items/1000 m^2^/day) than the average accumulation rates calculated for the others R_beach_ (15.9 items/1000 m^2^/day), making the R_beach_ of Malta not suitable for the analysis.

### Data compilation and analysis

For each of the 24 selected beaches a fixed 100 m portion of beach was defined. The coordinates of the starting point and the ending point of the portion were recorded and the distance between the two points was calculated with the following equation:

1 $$D = {\text{ cos}}^{{ - {1}}} ({\text{sin}}\left( {{\text{rad}}\left( {Lat_{S} } \right)} \right) \, + {\text{ cos}}\left( {{\text{rad}}\left( {Lat_{S} } \right) \, \times {\text{ cos}}\left( {{\text{rad}}\left( {Lat_{E} } \right)} \right) \, \times {\text{ cos}}\left( {{\text{rad}}\left( {Long_{S} - Long_{E} } \right)} \right)} \right) \, \times { 6371 } \times {1}000,$$

where *D* is the calculated distance in m, *Lat*_*S*_ is the starting latitude (in decimal degree), *Long*_*S*_ the starting longitude (in decimal degree), *Lat*_*E*_ the ending latitude (in decimal degree) and *Long*_*E*_ the ending longitude (in decimal degree). The functions cos^−1^ returns the arccosine of an angle, cos returns the cosine of an angle, sin returns the sine of an angle and rad converts degrees into radians. When *D* is not equal to 100 m (most of the cases) the number of items collected on the portion were corrected for the distance in order to normalize all the data for all the beaches. The following equation was used to correct the data:

2 $$NI_{cor} = NI_{col} \times { 1}00 \, /D,$$

where *NI*_*cor*_ is the number of items corrected for distance, *NI*_*col*_ the number of items collected on the fixed portion of beach and *D* the calculated distance from Eq. (1). Note that the number of items corrected for distance is only used to show the number of items per 100 m of beach: it is not used for the calculation of the accumulation rates or the accumulation index.

Finally, for each beach, the surface of the 100 m fixed portion was estimated by using the “Polygon” tool from Google Earth. For each beach, the considered surface was measured from the water line to the back of the beach, between the starting and the ending points of the portion.

The accumulation rates of the marine litter is used here to estimate the accumulation of marine litter or of a given item per unit of surface and per unit of time. In this study the accumulation rates are given in number of items/1000 m^2^/day. It is calculated as follows:

3 $$AR = \, \left( {NI_{col} /S/T} \right) \, \times { 1}000,$$

where *AR* is the accumulation rate in number of items/1000 m^2^/day, *NI*_*col*_ the number of items collected on the fixed portion of beach (not corrected for the distance), *S* the surface of the fixed portion of beach in m^2^ and *T* the time elapsed between the survey and the last cleaning activity in days. The time elapsed since the last cleaning corresponds to the number of days elapsed since the last cleaning performed by the local authorities (or the previous survey when no cleaning activities were conducted by the local authorities). The AR of the MPs and pellets were always calculated by taking into account the time elapsed between each survey and not the time elapsed since the last cleaning, as due to their small size we assumed these particles cannot be removed even with mechanical cleaning. Moreover, for the very first survey performed on each beach, the AR of the MPs and the pellets were arbitrarily calculated by using the number of days elapsed between the survey and the 1st of January 2017 as it was not possible to estimate since when these particles were accumulating on the beaches. Then the average number of days used for the calculation was 83.6, 83.6 and 87.3 respectively for the T_beach_, the L_beach_ and the R_beach_.

The accumulation index (AI) was developed for this study. This index takes into account the accumulation rates of the marine litter and can be calculated as follows:

4 $$AI = {\text{ log}}_{{{1}0}} \left( {AR \times { 1}000} \right)$$

where *AI* is the accumulation index and *AR* the accumulation rate (see Eq. 3) in number of items/1000 m^2^/day.

### Composition of the marine litter

The composition of the marine litter was estimated by grouping the items into nine different categories, following the EU MSFD TG10 “Guidance on Monitoring of Marine Litter in European Seas”^53^: artificial polymer materials, rubber, cloth/textile, paper/cardboard, processed/worked wood, metal, glass/ceramics, unidentified and chemicals. No matter the type of beach considered or the month of the monitoring, the marine litter collected was largely dominated by the artificial polymer materials category (Supplementary Fig. S2). On average, this category represents for the T_beach_, L_beach_ and R_beach_, respectively 87.9% ± 3.1%, 97.3% ± 1.8% and 85.9% ± 11.8% of the items collected during the low season, and respectively 87.7% ± 8.6%, 95.9% ± 4.0% and 88.1% ± 5.3% of the items collected during the high season.

### Seasonality

To make the distinction between the low and the high touristic season, the number of visitors welcomed in each island in 2017 was used, with the exception of the island of Rab (Croatia) where only the data for 2016 are available (Supplementary Table S1). These data were normalized (standard score method) in order to highlight the low and high seasons (Fig. 1).

### Pilot actions implementation

In 2019, both the T_beach_ and L_beach_ of Sicily, Malta, Crete, Rhodes and Cyprus, and the T_beach_ of Mallorca were selected to carry out pilot actions in order to reduce the amount of waste that enters the environment through beaches. These activities were implemented during the summer 2019 and consisted in, amongst other, awareness campaigns directly on sites (i.e., on beaches or the area surrounding them) or through social media or radio, installation of new trash bins for mixed or recyclable waste, or the installation of new signs on existing bins. A complete description of the pilot actions can be found here^46^.

### Extrapolation for all Mediterranean islands

In order to extrapolate the accumulation of marine litter for all the Mediterranean island beaches, we proceed as follows: first the total coastline length of each of the eight islands was extracted from the “CCM River and Catchment Database”, JRC/IES (https://agrienv.jrc.it/activities/catchments/) with ArcGIS. Then the total length of each island’s beaches was measured using the “path” tool from Google Earth (Supplementary Table S2). On average, the beaches represent about 34.0% ± 11.9% of the coastline. This proportion is in agreement with previous study for the Mediterranean Sea^47^, as 54% of the coastlines are rocky and the remaining 46% include beaches, dunes, reefs, lagoons, estuaries and deltas.

During the peak of the high season (July–August), the average AR of marine litter of the ST category, of the MePs and of the MPs taken together represent 1028.4 items/100 m/day, 798.3 items/100 m/day and 57.7 items/100 m/day respectively for the T_beach_, L_beach_ and R_beach_. This represents an average of 628.1 ± 507.2 items/100 m/day.

We estimated that beaches of Mediterranean islands account for 6466.5 km ± 2268.0 km and that the items left on beaches by the visitors could account for 40.6 10^6^ ± 11.5 10^6^ items/day.

## Supplementary information

Supplementary Information.

## Data Availability

The seasonal marine litter abundances and accumulation rates dataset used in this work are available at https://doi.org/10.1594/PANGAEA.923731.
