# Supplementary material for: The generation of marine litter in Mediterranean island beaches as an effect of tourism and its mitigation
Source: Sci Rep. 2020 Nov 23;10:20326. doi: 10.1038/s41598-020-77225-5 (PMC7683737; doi:10.1038/s41598-020-77225-5)
Supplement: Supplementary file 1 — Supplementary Information. [file 41598_2020_77225_MOESM1_ESM.docx]

Supplementary information to *The generation of marine litter in Mediterranean island beaches as an effect of tourism and its mitigation*

Michaël Grelaud^1*^ and Patrizia Ziveri^1,2^

^1^Institute of Environmental Science and Technology (ICTA), Universitat Autònoma de Barcelona (UAB), Bellaterra, Spain

^2^ Catalan Institution for Research and Advanced Studies (ICREA), Barcelona, Spain

*Corresponding author: [michael.grelaud@uab.cat](mailto:michael.grelaud@uab.cat)

**
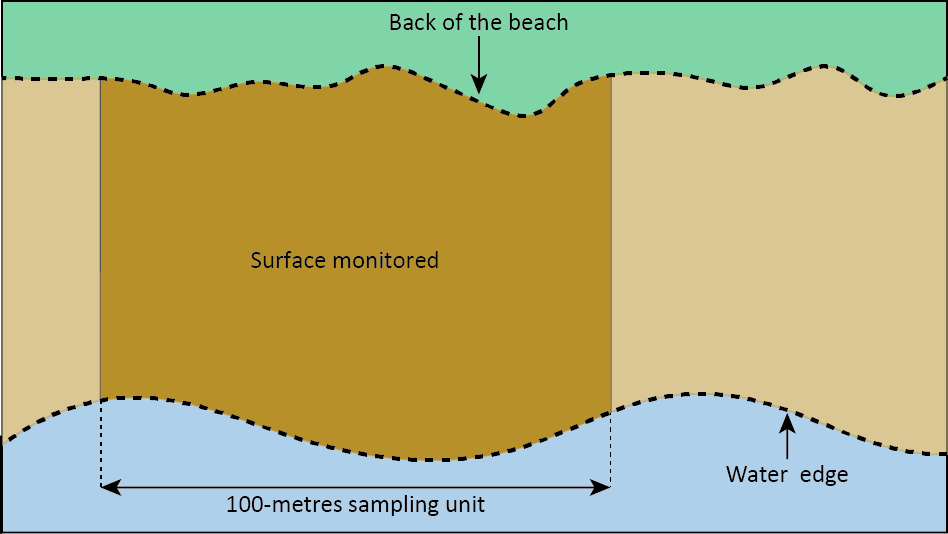
**

**Figure S1:** schematic drawing of the fixed 100m portion of beach monitored.


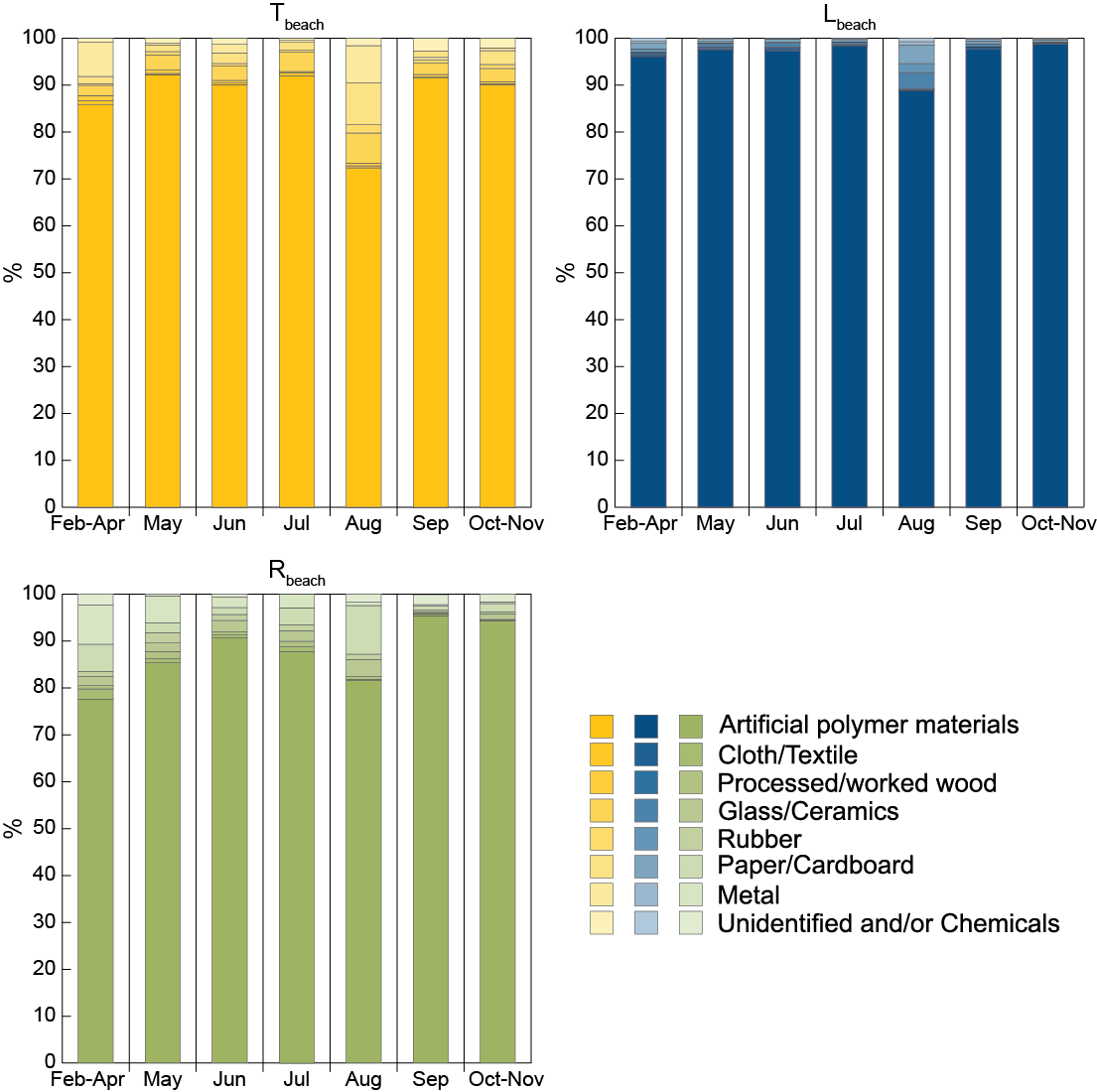


**Figure S2:** Composition of the marine litter for the T_beach_ (yellow), L_beach_ (blue) and R_beach_ (green).


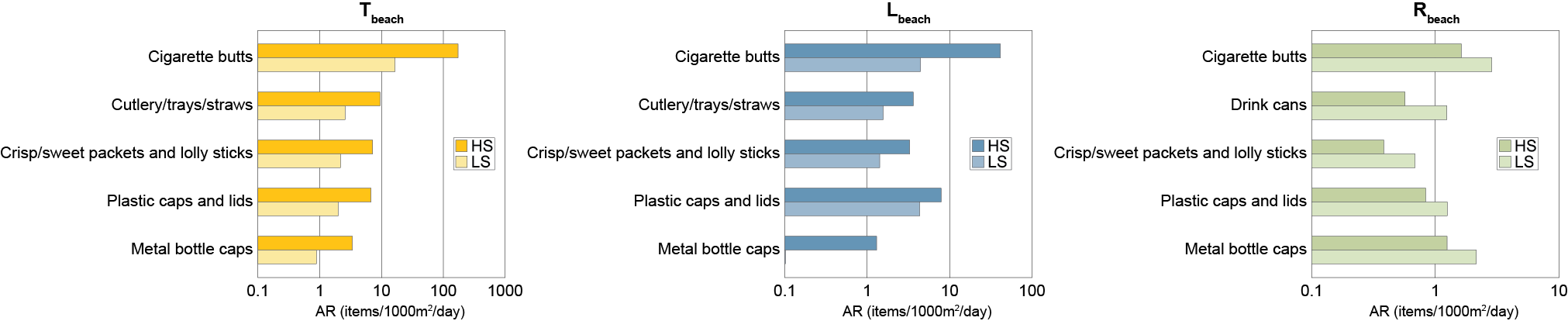


**Figure S3:** Comparison of the seasonal AR (logarithmic scale) of the 5 most abundant items collected on the 3 different types of beach in 2017.


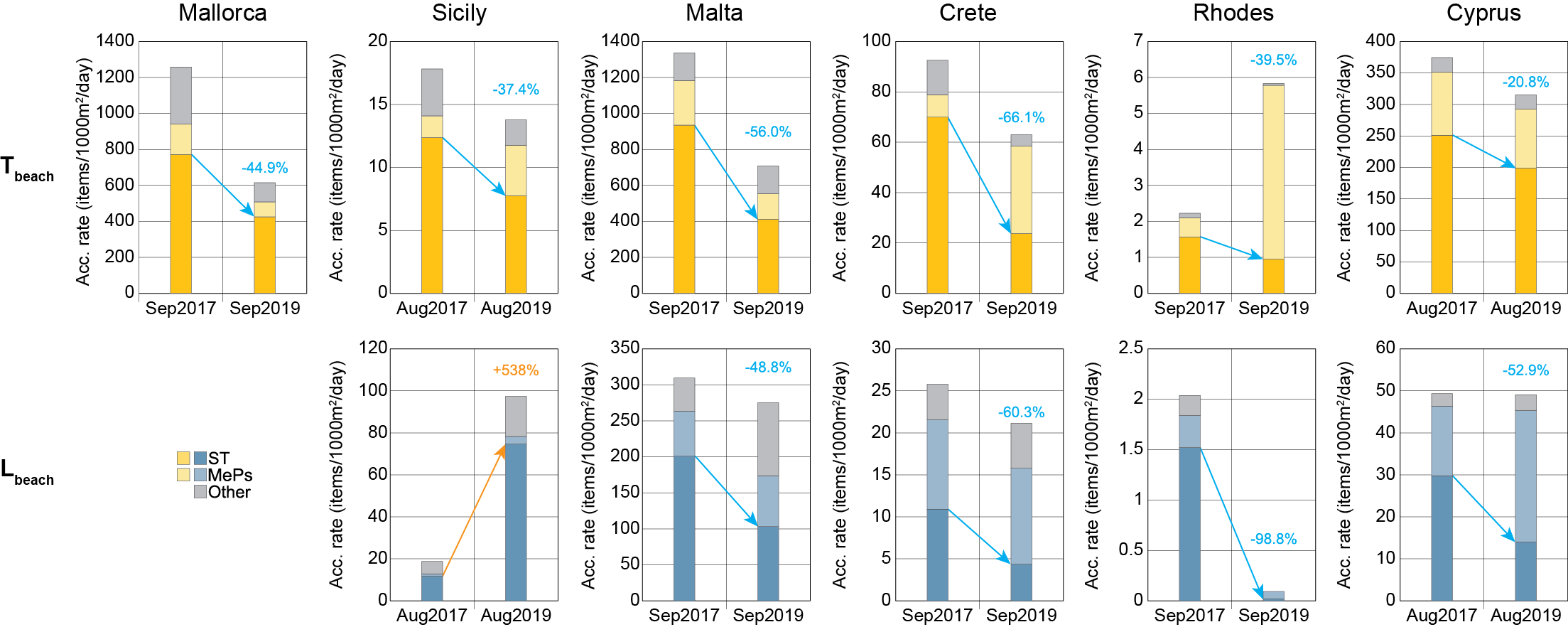


**Figure S4:** Comparison of the AR of the items of the ST category, of the MePs and of all the others items (excluding the MPs and the pellets) between August/September 2017 and August/September 2019. The light blue (orange) percentages refer to the decrease (increase) observed after the implementation of the pilot actions of the AR of the items from the ST category.


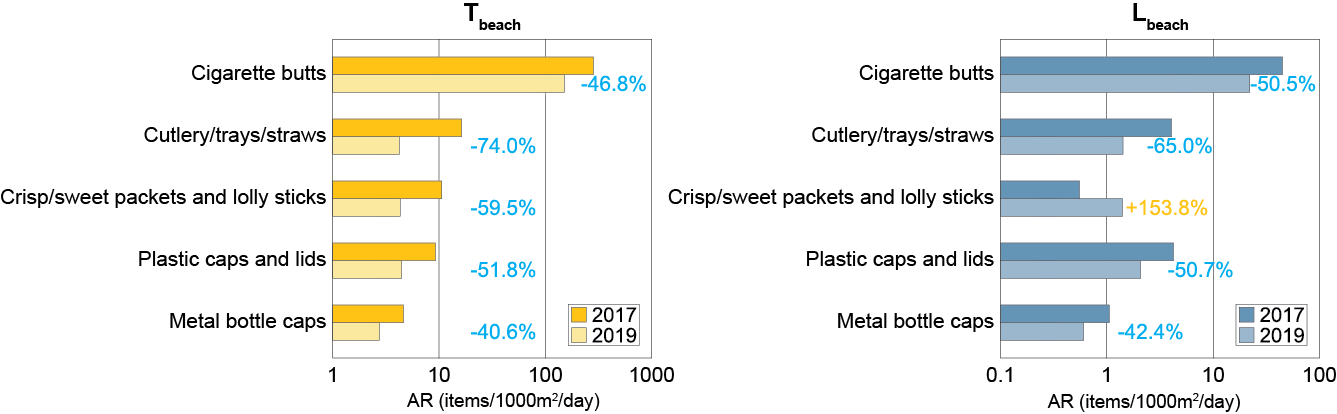


**Figure S5:** Comparison of the AR (logarithmic scale) of the five most abundant items of the ST category between August/September 2017 and August/September 2019 for the tested T_beach_ (left) and L_beach_ (right). The light blue (yellow) percentages refer to the decrease (increase) observed after the implementation of the pilot actions.

**Table S1:** Monthly number of visitors welcomed in each island in 2017 (2016 for Rab). MLC=Mallorca; SIC=Sicily; RAB=Rab; MLT=Malta; CRT=Crete; MYK=Mykonos; RHD=Rhodes and CYP=Cyprus. The data were collected from various sources: ^a^<http://www.caib.es>; ^b^<http://pti.regione.sicilia.it>; ^c^<https://www.dzs.hr>; ^d^<https://nso.gov.mt>; ^e^<http://www.insete.gr>; and ^f^<http://www.cystat.gov.cy>.

|  | MLC^a^ | SIC^b^ | RAB^c^ | MLT^d^ | CRT^e^ | MYK^e^ | RHD^e^ | CYP^f^ |
| --- | --- | --- | --- | --- | --- | --- | --- | --- |
| January | 319296 | 927740 | 121 | 104069 | 73303 | 4815 | 29142 | 62611 |
| February | 373625 | 855302 | 175 | 103899 | 71065 | 6123 | 26190 | 82209 |
| March | 593830 | 1081276 | 895 | 140922 | 90936 | 8582 | 32402 | 140873 |
| April | 1164850 | 1413243 | 2437 | 199586 | 309599 | 18372 | 121336 | 286331 |
| May | 1576818 | 1553782 | 8560 | 215373 | 591194 | 49811 | 282675 | 418732 |
| June | 1852504 | 1654988 | 21986 | 226333 | 790880 | 95036 | 409963 | 472450 |
| July | 2126395 | 1850267 | 59206 | 251066 | 974911 | 139099 | 508428 | 531030 |
| August | 2025142 | 1857638 | 59330 | 284844 | 930138 | 139869 | 497023 | 523651 |
| September | 1777922 | 1735506 | 16678 | 235881 | 784700 | 85832 | 410285 | 483716 |
| October | 1318210 | 1528290 | 3309 | 240113 | 480312 | 26710 | 233139 | 406870 |
| November | 412495 | 1037865 | 467 | 162530 | 80801 | 1366 | 29097 | 144676 |
| December | 402284 | 1094825 | 182 | 109222 | 72312 | 1692 | 28776 | 98924 |

**Table S2:** Total coastline and beach length for each island. MLC=Mallorca; SIC=Sicily; RAB=Rab; MLT=Malta; CRT=Crete; MYK=Mykonos; RHD=Rhodes and CYP=Cyprus.

| Island | Coastline (km) | Beaches (km) |
| --- | --- | --- |
| MLC | 427.39 | 51.59 |
| SIC | 1061.87 | 531.37 |
| RAB | 89.28 | 12.27 |
| MLT | 157.87 | 4.93 |
| CRT | 926.35 | 236.45 |
| MYK | 69.84 | 11.65 |
| RHD | 224.77 | 112.38 |
| CYP | 716.20 | 289.63 |
